# Empirical Comparison of Distributed Source Localization Methods for Single-Trial Detection of Movement Preparation

**DOI:** 10.3389/fnhum.2018.00340

**Published:** 2018-09-03

**Authors:** Anett Seeland, Mario M. Krell, Sirko Straube, Elsa A. Kirchner

**Affiliations:** ^1^Robotics Innovation Center, German Research Center for Artificial Intelligence (DFKI GmbH), Bremen, Germany; ^2^Robotics Group, Faculty of Mathematics and Computer Science, University of Bremen, Bremen, Germany; ^3^International Computer Science Institute, University of California, Berkeley, Berkeley, CA, United States; ^4^University of California, Berkeley, Berkeley, CA, United States

**Keywords:** source imaging, inverse problem, MRCP, brain-computer interface, EEG, movement detection

## Abstract

The development of technologies for the treatment of movement disorders, like stroke, is still of particular interest in brain-computer interface (BCI) research. In this context, source localization methods (SLMs), that reconstruct the cerebral origin of brain activity measured outside the head, e.g., via electroencephalography (EEG), can add a valuable insight into the current state and progress of the treatment. However, in BCIs SLMs were often solely considered as advanced signal processing methods that are compared against other methods based on the classification performance alone. Though, this approach does not guarantee physiological meaningful results. We present an empirical comparison of three established distributed SLMs with the aim to use one for single-trial movement prediction. The SLMs wMNE, sLORETA, and dSPM were applied on data acquired from eight subjects performing voluntary arm movements. Besides the classification performance as quality measure, a distance metric was used to asses the physiological plausibility of the methods. For the distance metric, which is usually measured to the source position of maximum activity, we further propose a variant based on clusters that is better suited for the single-trial case in which several sources are likely and the actual maximum is unknown. The two metrics showed different results. The classification performance revealed no significant differences across subjects, indicating that all three methods are equally well-suited for single-trial movement prediction. On the other hand, we obtained significant differences in the distance measure, favoring wMNE even after correcting the distance with the number of reconstructed clusters. Further, distance results were inconsistent with the traditional method using the maximum, indicating that for wMNE the point of maximum source activity often did not coincide with the nearest activation cluster. In summary, the presented comparison might help users to select an appropriate SLM and to understand the implications of the selection. The proposed methodology pays attention to the particular properties of distributed SLMs and can serve as a framework for further comparisons.

## 1. Introduction

Suffering a stroke nowadays often means lifelong impairments in daily living. Especially the upper limb recovery rate is not satisfactory, given that over 60% of the patients still have dysfunctions 6 month post-stroke (Kwakkel et al., 2003). Further, the number of yearly new cases of stroke will rise in Europe from 613, 184 in 2015 to 819, 771 in 2035 mostly due to demographic changes (Stevens et al., 2017). The high importance for the society to improve recovery is one of the driving forces for the development of new technologies, like brain-computer interfaces (BCIs), that allow by means of decoding brain activity to reconstruct the functional loop between brain, motor and sensor system (e.g., Muralidharan et al., 2011).

The majority of BCIs (for review see Nicolas-Alonso and Gomez-Gil, 2012) or more implicit approaches like embedded brain reading (eBR) (Kirchner and Drechsler, 2013; Kirchner et al., 2013b, 2014), neuroergonomics (Mehta and Parasuraman, 2013) and passive BCIs (Zander and Kothe, 2011) rely on the electroencephalogram (EEG) to measure brain activity due to its non-invasiveness, high temporal resolution and low operational costs. In the implicit approaches, detailed information must be extracted from the *natural* EEG to infer on the intention of the human (see Kirchner et al., forthcoming for discussion). Usually, machine learning algorithms are applied to EEG single-trials to detect, e.g., movement preparation in advance of the intended movement onset. This knowledge can be integrated in technically assisted neuro-motor rehabilitation (Kirchner et al., 2013a). In this way, even in the early therapy after stroke, goal-directed movements of the affected arm can be trained. Although there might be too little muscular activity, just the detection of the intented movement from brain activity can trigger a robotic device, like an orthosis or exoskeleton, to perform the movement (Monge-Pereira et al., 2017). To further significantly induce cortical plasticity, the detection of an intent should occur on average at least 50 ms prior to the subsequent activation of the external device (Mrachacz-Kersting et al., 2012). Hence, EEG data needs to be processed within a millisecond range. Additionally, spatial information about the brain activity in this context can encode the moving body part. Penfield and colleagues proposed the first somatotopic map of the human primary motor cortex (Penfield and Boldrey, 1937; Penfield and Rasmussen, 1950) obtained from electrical stimulation. Up to now, this mapping has extensively been studied, also with non-invasive high resolution neuro-imaging methods, and a lot of evidence has accumulated that distinct brain regions for the body parts exist despite an overlap (e.g., Meier et al., 2008; Plow et al., 2010). Collectively, single-trial decoding to support neuro-motor rehabilitation has time requirements, and can benefit in particular from an increased spatial resolution.

From the therapist's point of view it would be further beneficial to know what the decoding is based on. For example, there is evidence that in the early stage after stroke the ipsilateral hemisphere shows increased activity, and a “normal” contralateral activity pattern before movement onset is absent (Yilmaz et al., 2015). During the recovery process the contralateral pattern may return (Yilmaz et al., 2015). Hence, monitoring the changes in the decoding strategy (favoring ipsilateral or contralateral parts of the motor cortex) can give valuable insights in the progress of recovery. As a more advanced step the decoding algorithm may be further guided by therapeutical knowledge to use only specifc regions of the brain. This can be also an effective appoarch for neurofeedback therapy (Micoulaud-Franchi et al., 2015). A prerequisite to exploit these possibilities is a transformation from sensor space to brain space inside the interface.

Both, a transformation from sensor to brain space and an increased spatial resolution of the EEG can be achived by reconstructing its underlying sources. Methods following this approach are source localization methods (SLMs). So far, SLMs showed promising results in increasing the precision of single-trial classification (e.g., Kamousi et al., 2007; Cincotti et al., 2008; Yuan and He, 2009; Besserve et al., 2011; Edelman et al., 2016; Wronkiewicz et al., 2016). SLMs model the sources of the EEG in the cortex and the physical properties of the head volume conductor. In this way, the relation between a source pattern and the corresponding scalp potential distribution can be established. However, this relation is not bijective: there are infinitely many possible source patterns for a measured potential distribution at the scalp. Hence, beside the aforementioned modeling assumptions, additional constraints have to be set to solve the so-called *inverse problem* uniquely. In the BCI-literature, most of the studies favor the linear distributed approach (e.g., Ilmoniemi, 1993; Wang et al., 1993; Hämäläinen and Ilmoniemi, 1994; Pascual-Marqui et al., 1994; van Veen et al., 1997; Dale et al., 2000) for the inverse problem due to its low computational needs. Distributed SLMs assume a fixed number of sources in the order of thousands that are usually distributed uniformly with fixed orientations over the solution space (e.g., gray matter). In this way, only the source strengths have to be estimated. Nevertheless the problem is highly underdetermined, so additional constraints are introduced varying from purely mathematical to physiologically motivated assumptions (Michel et al., 2004). Due to the necessity of additional constraints and the fact that the EEG and its underlying sources are not fully understood yet, new SLMs are continuously developed (Pizzagalli, 2007; Grech et al., 2008; Becker et al., 2015). For BCIs, mainly the well-known, most established SLMs have been applied, like minimum norm (Noirhomme et al., 2008; Besserve et al., 2011; Edelman et al., 2014; Wronkiewicz et al., 2015, 2016), weighted minimum norm (Qin et al., 2004; Babiloni et al., 2007; Kamousi et al., 2007; Cincotti et al., 2008; Yuan and He, 2009; Goel et al., 2011; Edelman et al., 2015, 2016), standardized low resolution electromagnetic tomography (Congedo et al., 2006; Lotte et al., 2009; Handiru et al., 2017), local autoregressive average (Menendez et al., 2005; Poolman et al., 2008) and beamformer methods (Grosse-Wentrup et al., 2009; Ahn et al., 2012). However, a comparison of different distributed SLMs has rarely been reported so far. Often one SLM has been applied in one publication without giving a reason for its selection. Therefore, we empirically compare distributed SLMs in this paper. Our selection of SLMs is in line with what has been used so far in BCIs: We compared the well-established methods weighted minimum norm (wMNE), standardized low resolution electromagnetic tomography (sLORETA), and dynamic statistical parameter mapping (dSPM). All three methods are available as open-source, e.g., via the software Brainstorm (Tadel et al., 2011).

Because the classification performance is of high importance for the interface, most of the design choices between various algorithms inside the signal processing chain are made based on this metric. However, the optimal algorithm selected in this way does not have to be physiological meaningful or interpretable, at least not without further effort (e.g., Krell and Straube, 2017). Since the aim of SLMs is to reveal the current density distribution underlying the EEG, SLMs cannot only be used to increase classification performance but also to interpret results physiologically. In the bio-imaging field of research, where most of the SLMs have been developed, the most common metric to measure the quality of SLMs is a distance metric. It describes how far the reconstructed sources are away from the *true* source distribution. For this metric, which is usually measured to the source position of maximum activity, we propose a variant that is better suited for the single-trial case in which several sources are likely and the actual maximum is unknown. For example, the neural process of interest might be superimposed during several single-trials by other neural correlates. These additional sources might be stronger in amplitude than the expected ones. Also artifacts, like eye blinks, can result in much higher amplitudes than the signal of interest. Further, considering *real-world* single-trial data, it is almost impossible to define a complete ground truth source distribution. If therefore the ground truth is reduced to a small set of expected activity regions, it is tolerated that further sources are reconstructed even if these sources have a larger amplitude. Last but not least, it has been argued that due to the underdetermined nature of distributed SLM, over- and underestimation of source strengths can easily occur (Grave de Peralta-Menendez and Gonzalez-Andino, 1998; Michel et al., 2004; Wendel et al., 2009). Hence, our distance metric has relaxed requirements regarding accurate amplitudes.

In this paper, we compare three SLMs with the aim to use one in a real-time single-trial detection task of movement preparation. Hence, the classification performance was a criterion for the comparison. In addition, we calculated a distance measure to evaluate the physiological plausibility of the reconstructed sources. For that, we expect the methods to extract at least one source in a subarea of the primary motor cortex (reference region). In this way, the localization error was measured in each movement preparation trial. This alternative distance metric is obtained by clustering the current density distribution and measuring the distance from the reference region to the nearest activation cluster. The metric can also be normalized to account for different numbers of clusters. Results differed depending on the metric. While no significant differences between the SLMs were obtained in terms of classification performance, our distance metric favored wMNE. Thus, the presented comparison represents a case where conclusions might not be drawn based on the classification performance alone. The superiority of wMNE is based on the distance metric calculation that uses clusters and an *a priori* defined reference region. That means, our results indicate that wMNE can be the method of choice, i.e., has the smallest distance, when compared to an expected source location.

The paper is organized as follows: section 2 describes the empirical data and reviews the SLMs that were compared. In section 3 the methodology for the comparison is presented. Results of the comparison as well as a discussion can be found in sections 4 and 5, respectively. Finally, section 6 concludes the work and gives an outlook.

## 2. Materials and foundations

### 2.1. Data

We considered empirical data that has been acquired under highly controlled conditions with only one type of movement. In this way, the data is more realistic in comparison to simulations, but it is still possible to make reliable expectations about a source in the primary motor cortex (see section 3.2). The data was previously recorded at our lab and has been described in detail in Tabie and Kirchner (2013). Eight right-handed healthy male volunteers (19–32 years old) gave written consent to participate in the study, that was approved by the local ethics committee of the University of Bremen and in accordance with the Declaration of Helsinki.

#### 2.1.1. Paradigm

Experiments took place in a dimly lit shielded cabin. Participants sat in a comfortable chair behind a table, resting their right arm on the table. The right hand was placed on a flat switch representing the *resting* condition. The task comprised performing arm movements as fast as possible from the flat switch rightwards to a buzzer approximately 20 cm away and then returning to the resting position. There was no command nor cue to start a movement. Thus, movements were voluntarily initiated by the subjects. Subjects were instructed to avoid eye-movements by fixating a cross on a screen during the experiment. In addition, negative feedback was given to the subjects whenever the *resting* condition lasted less than 5 s. Such invalid trials were not considered for analyses. A complete experiment consisted of 120 valid movements split in three runs with short breaks in between.

#### 2.1.2. Recorded data streams

One hundred and twenty-eight electrodes arranged according to the extended 10–20 system were used for EEG data acquisition (acti-CAP; Brain Products GmbH, Munich, Germany) and impedance was kept below 5 kΩ. The EEG with reference at FCz was recorded at 5 kHz using four BrainAmp DC amplifiers (Brain Products GmbH, Munich, Germany). Together with the EEG data, the electromyogram (EMG) was recorded from four muscles of the left and right arm using a bipolar setup and a BrainExG MR amplifier. Before storing the data on disk an analog band pass filter between 0.1 and 1 kHz was applied. In addition, events from the flat switch and the buzzer, i.e., pressing and releasing, were marked in the EEG/EMG data stream.

A second data stream comprised motion tracking data of a passive infrared marker mounted on the right hand. As motion tracking system three ProReflex 1000 cameras (Qualisys AB, Gothenburg, Sweden) were used. The data was acquired at 500 Hz and the start and end of the stream were marked in the EEG for later synchronization.

### 2.2. Preprocessing

From the acquired data, only the EEG and the motion tracking data were analyzed further. All EEG data were used, i.e., no data segments were skipped because of artifacts, to mimic the application case. In the following, the steps in preparing the analyses are described.

#### 2.2.1. Movement onset

The movement onset defines time point zero in the later analyses. It also refers to the latest point in time when a prediction about an upcoming movement can be made. The motion tracking data was used to obtain the movement onset in each trial. After synchronization with the EEG stream, the tracking data was analyzed backwards in time starting at the release events of the flat switch. For each trial, a movement onset marker was added to the EEG stream, when the speed per sampling interval crossed a threshold of 0.075 mm/ms which corresponded to the precision of the motion tracking system.

#### 2.2.2. Reduction of irrelevant components

Movement preparation and execution are reflected in the EEG for example by movement related cortical potentials (MRCPs) (Deecke et al., 1976; Shibasaki and Hallett, 2006). MRCPs describe slow changes in the amplitude starting about 2 s before movement onset (Stančák et al., 2000; Shibasaki and Hallett, 2006). To capture this slow change, the frequency spectrum as well as the dimension of the data can be reduced. Hence, the high sampling frequency provided by the hardware (5 kHz) was decimated to 20 Hz in two steps using anti-alias finite impulse response filters (Crochiere and Rabiner, 1975). After the first step the intermediate sampling rate resulted in 100 Hz. In the second step, the filter was further parameterized to attenuate all frequencies higher than 4 Hz. In addition low frequency components close to the direct current offset were removed by an infinite impulse response filter.

### 2.3. Source localization

In distributed SLMs a linear model of the data is considered (e.g., Wendel et al., 2009):

(1) $$\begin{array}{l} d=Ls+n. \end{array}$$

Here, *d* refers to the measured data as a vector of *N*_e_ components corresponding to *N*_e_ electrodes, *L* denotes the (*N*_e_×3*N*_s_)-lead field matrix that contains the relation of source activations in the three Cartesian directions to the electrode measurements, *s* corresponds to the 3*N*_s_ true source activations, and *n* models some additive noise in the sensor space.

First, the *forward model* *L* has to be computed. The computation of *L* incorporates the head geometry and conductivity values (ranging from simple nested spheres to complex boundary or finite element models), the electrode positions as well as the chosen source model (positions, orientation constraints). Then, solving the *inverse problem* can be described by Grech et al. (2008)

(2) $$\begin{array}{l} \underset{ŝ}{\min}||d-Lŝ|{|}^{2}+\alpha R\left(ŝ\right) \end{array}$$

where ŝ denotes a vector of 3*N*_s_ estimated source activations, α denotes the regularization parameter, and *R*(ŝ) represents a regularization function that differs for the respective algorithm. The first term minimizes the least squares error between data and the transformation of the sources to the data space.

Brainstorm Version 17-Sep-2015 (Tadel et al., 2011) was used to compute the solution for three SLMs: weighted Minimum Norm Estimate (wMNE), dynamic Statistical Parametric Mapping (dSPM) and standardized low resolution electromagnetic tomography (sLORETA). Among the chosen methods, it has been shown that sLORETA achieves zero dipole localization error for a single active source (Pascual-Marqui, 2002). However, we assume that it is very likely that several sources are active, especially when single-trials are considered. Thus, also other methods which perform worse on single source localization are investigated. The three SLMs are described in more detail in the following. All used parameters for the methods, parameter optimization settings as well as other components for source localization (head model, etc.) were kept fixed for comparison and can be found in Table 1.

**Table 1:** Parameters for source localization components.

**Source model**	
Geometry	Brain surface of ICBM152 template (Fonov et al., 2011), 15, 002 vertices
Orientation constraints	No
**Head model**	
Geometry	3-shell nested sphere model (Berg and Scherg, 1994; Zhang, 1995)
Radii	Brain: 8.3 cm, skull 8.8 cm, skin 9.4 cm
Conductivity	Brain: 0.33 S/m, skull: 0.0042 S/m, scalp: 0.33 S/m
Electrode positions	Standard positions of tde extended 10-20 system, mapped on tde scalp
**Inverse metdod**	
Noise covariance	Based on concatenated data during resting condition; Tikhonov regularized witd ϵ = 0.1
Regularization parameter α	Optimized with generalized cross validation (Hansen, 1998, 2007); if optimized α < 0.01, α is set to the default value of 1/3
Depth weighting	Maximal amount of 10 with an order of γ = 0.5; applied for wMNE and dSPM methods

#### 2.3.1. wMNE

This algorithm is an improvement of the classical minimum norm approach (Hämäläinen and Ilmoniemi, 1994), where the solution with lowest overall intensity is selected, i.e., *R*(ŝ) = ||ŝ||^2^. The classical approach prefers superficial sources since for the same scalp potential distribution the strength of deeper sources has to be much higher compared to sources close to the electrodes (e.g., Fuchs et al., 1999; Lin et al., 2006). To account for the bias toward superficial sources, different weighting strategies have been proposed (Grech et al., 2008), among others a weighting that normalizes the columns of the lead field matrix (Jeffs et al., 1987; Baillet et al., 2001; Lin et al., 2006). In this case the three diagonal elements of *W* for source point *p* can be computed by

(3) $$\begin{array}{l} w_{p}={\left(\underset{i\in I_{p}}{\sum }l_{•i}{\left(l_{•i}\right)}^{T}\right)}^{-\gamma }\;\text{for }1\leq p\leq N_{\text{s}}, \end{array}$$

where *I*_*p*_ contains all indices that belong to point *p* and *l*_•*i*_ refers to the *i*th column of *L*. The order of depth weighting can be controlled with the parameter γ. Each column of *L* contains the topography of a source, i.e., the signal that ends up at the electrodes when this source would be active. Thus, the column norm of a superficial source is greater than the column norm of a source far away from the electrodes. This weighting is integrated into *R*, i.e., *R*(ŝ) = ||*Wŝ*||^2^, and finally a solution for Equation (2) is given by

(4) $$\begin{array}{l} ŝ=G_{\text{wmne}}d \end{array}$$

where *G*_wmne_, the inverse operator of the weighted minimum norm algorithm, is

(5) $$\begin{array}{l} G_{\text{wmne}}={\left(W^{T}W\right)}^{-1}L^{T}{\left(L{\left(W^{T}W\right)}^{-1}L^{T}+\alpha C_{\text{n}}\right)}^{-1}. \end{array}$$

Here, spatial correlations of the different electrodes are modeled by the noise covariance matrix *C*_n_.

#### 2.3.2. dSPM

dSPM (Dale et al., 2000) is computed based on the minimum norm or weighted minimum norm inverse operator by normalizing its rows. For normalization, the source estimates of the noise are computed based on the noise covariance matrix *C*_n_. These estimates form a diagonal normalization matrix *S*_dspm_ with elements

(6) $$\begin{array}{l} s_{p}^{\text{dspm}}={\left(\underset{i\in I_{p}}{\sum }g_{i•}C_{\text{n}}g_{i•}^{T}\right)}^{-1/2}\;\text{for }1\leq p\leq N_{\text{s}}, \end{array}$$

for the three rows of *G* that belong to the source point *p*. The inverse operator of the dSPM algorithm is then given by

(7) $$\begin{array}{l} G_{\text{dspm}}=S_{\text{dspm}}G. \end{array}$$

In our empirical evaluation, we selected the wMNE inverse operator as *G*.

#### 2.3.3. sLORETA

A different normalization strategy is pursued within the sLORETA algorithm (Pascual-Marqui, 2002). Here, a second source of variance beside the measurement noise is considered: the variance of the actual sources. It has been shown that this normalization can be derived from the resolution matrix *A* = *GL* (Pascual-Marqui, 2002). Thus, the blocks $s_{p}^{\text{sloreta}}$ of the block diagonal normalization matrix *S*_sloreta_ are given by

(8) $$\begin{array}{l} s_{p}^{\text{sloreta}}={\left(A_{\left[i\in I_{p},j\in I_{p}\right]}\right)}^{-1/2}\;\text{for }1\leq p\leq N_{\text{s}}, \end{array}$$

where *A*_[*i*∈_*I*__*p*_, *j*∈*I*_*p*_]_ denotes the (3 × 3)-diagonal block of the resolution matrix corresponding to source point *p*. Again, the inverse operator of the sLORETA algorithm is finally obtained by

(9) $$\begin{array}{l} G_{\text{sloreta}}=S_{\text{sloreta}}G, \end{array}$$

where *G* is usually the MNE inverse operator.

## 3. Criteria of comparison

This section presents our suggested methodology for comparing SLMs for single-trial classification. To determine which SLM is most effective for movement prediction, all were integrated in our signal processing scheme and the classification performance was calculated. A further rationale was to provide insights into the impact of the different SLMs when interacting with other components like a classification algorithm. Since the aim of SLMs is to reveal the current density distribution underlying the EEG, SLMs can be used to interpret results physiologically. Hence, the general plausibility of the reconstructed source distribution is assessed by computing the distance to a reference region obtained from literature.

In the following, the two metrics are explained for the concrete example of movement prediction, i.e., the classification between *resting* and *movement preparation*. Nevertheless it is possible to apply the underlying methodology to other data and classification problems. Thus, section 3.3 describes possible generalizations.

### 3.1. Classification performance

The classification performance evaluates the whole processing chain for movement prediction that consisted of preprocessing (see section 2.2), segmentation, feature extraction based on source localization, and classification. Except the source localization step, similar processing chains were already successfully utilized in former studies (Folgheraiter et al., 2011; Seeland et al., 2013, 2015; Kirchner et al., 2014; Wöhrle et al., 2014; Straube et al., 2015). The framework pySPACE (Krell et al., 2013) was used for processing and performance computation. In the following, the processing starting from the segmentation step is described and subsequently details about the performance metric are given.

First, time series segments of 0.2 s length (four samples) were extracted and labeled depending on the movement onset event: Segments between 3 to 2 s before movement onset belonged to the *resting* class and segments ending at −0.05 s belonged to the *movement preparation* class. Next, data of each subject were split into training and testing data following a 3-fold cross validation scheme, where each fold corresponded to one of the three experimental runs (section 2.1.1). The computation of the inverse operators was performed on the training data: For noise covariance estimation the *resting* class data were concatenated and for optimization of α the measurement data at −0.05 s were considered. As next step, a dimensionality reduction to the most promising sources was required to avoid overfitting of the classifier and to reduce computational load. Hence, the inverse operators were reduced to the 750 source positions that showed highest activity on the training data. No other dimensionality reduction method was applied for consistency reasons since the selected positions were the same to those used in the distance calculation (section 3.2). For each of the four time points the strength of activation in each direction was chosen as a feature. Hence, in total 9,000 features were used (750 × 3 × 4 = 9, 000). Feature normalization coefficients were also computed on the training data for each dimension in order to normalize features to have zero mean and a standard deviation of one. Subsequently, a linear support vector machine (SVM) was trained (Chang and Lin, 2011). The unbalanced class ratio of 1:5 for *movement preparation* and *resting* examples was mitigated by setting the class weight parameter of the underrepresented class to 2. Finally, the threshold of the SVM was optimized based on the training data. In order to optimize the regularization parameter λ of the SVM, training and threshold optimization were performed inside a nested 2 × 5-fold cross validation. In each cross validation iteration six values of λ were tested (λ ∈ {10^−6^, 10^−5^, …, 10^0^}) and the one with the highest classification performance was selected.

Performance of movement prediction was assessed on the testing data. As metric the balanced accuracy (BA) was computed (Straube and Krell, 2014), which refers to the arithmetic mean of the true positive rate and true negative rate.

### 3.2. Distance to reference region

While the computation of the classification performance is rather straightforward, the distance metric in conjunction with empirical data brings along several challenges. Unlike in simulation, the number of actual sources and their amount of activation is not known. It is even unrealistic to assume only active sources correlated to movement preparation, especially at single-trial scale or in unrestricted application scenarios. Further, the inherent disability of distributed SLMs to estimate the amplitudes of the sources correctly under these conditions makes it problematic to assess distributed SLMs solely based on maximum amplitudes. To address these challenges, we made the assumption that activity in the primary motor cortex close before the movement onset is reliable. Instead of comparing this reference to the point of maximum activity of the reconstructed source distribution, which is typically done e.g., for computation of the dipole localization error but also other metrics (Bai et al., 2007; Molins et al., 2008; Hauk et al., 2011), we generated clusters from 5% of the solution space, i.e., 750 vertices, with highest activations. In this way, small errors or fluctuations in amplitude estimation are considered and have much less influence on the results. Then, the distances of all clusters and the reference region were calculated and the smallest distance was reported. However, this procedure introduced a bias into the metric: a source distribution with a large number of clusters will have a higher probability to return a small distance to the reference region than a distribution with a small number of clusters, independent of the correctness of these distributions. Hence, besides reporting the number of computed clusters side by side to the distance, the distance was normalized with the average smallest distance a random source distribution with the corresponding number of clusters would achieve. In the following, each step in the metric calculation process is described in detail.

#### 3.2.1. Reference region

During our experiments (see section 2.1.1), subjects were requested to perform movements which mainly involved an external rotation of the right elbow. Hence, coordinates of maximal activity during right elbow movements were extracted from the literature (Grafton et al., 1993; Lotze et al., 2000; Alkadhi et al., 2002). The extracted Talairach coordinates were converted into MNI coordinates (Lancaster et al., 2007; Radua and Mataix-Cols, 2015) and mapped to vertices in the source space (see Table 2, reference coordinates). Next, a reference region was constructed that included *all* reference coordinates. For that, all vertices along the shortest path between the reference coordinates were added. Subsequently, the region was increased by all neighbors of the vertices on the shortest path. Using a region also accounted to some degree for inter-individual differences. The reference region, that consisted in total of 21 vertices, is depicted in Table 2 on the right.

**Table 2:** Determination of the reference region.

		**Talairach coordinates**			**MNI coordinates**			**Reference coordinates**			
**Study**	**Method**	***x***	***y***	***z***	***x***	***y***	***z***	***x***	***y***	***z***	
**Grafton et al., 1993**	**PET**	**−22**	**−22**	**60**	**−22**	**−17**	**65**	**−16.6**	**−17.8**	**65.8**	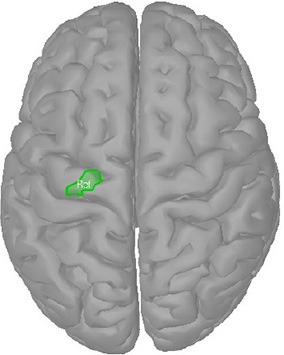
**Lotze et al., 2000**	**fMRI**	**−28**	**−24**	**64**	**−28**	**−19**	**69**	**−28.0**	**−24.6**	**70.0**	
**Alkadhi et al., 2002**	**fMRI**	**−29**	**−25**	**61**	**−29**	**−20**	**66**	**−28.3**	**−24.3**	**62.9**	
		**−29**	**−26**	**62**	**−29**	**−21**	**67**	**−28.0**	**−24.6**	**70.0**	

***Left**: Talairach coordinates of maximum activation during right elbow movements. An MNI-to-Talairach transformation (Lancaster et al., 2007) was applied to convert Talairach to MNI coordinates (Radua and Mataix-Cols, 2015). Finally, the reference coordinates, i.e., the closest points in the source space to the MNI coordinates, were calculated.**Right**: Reference region mapped onto the cortex surface of the ICBM152 template, i.e., the source space for source localization*.

#### 3.2.2. Clustering of activations

**Figure 1:**
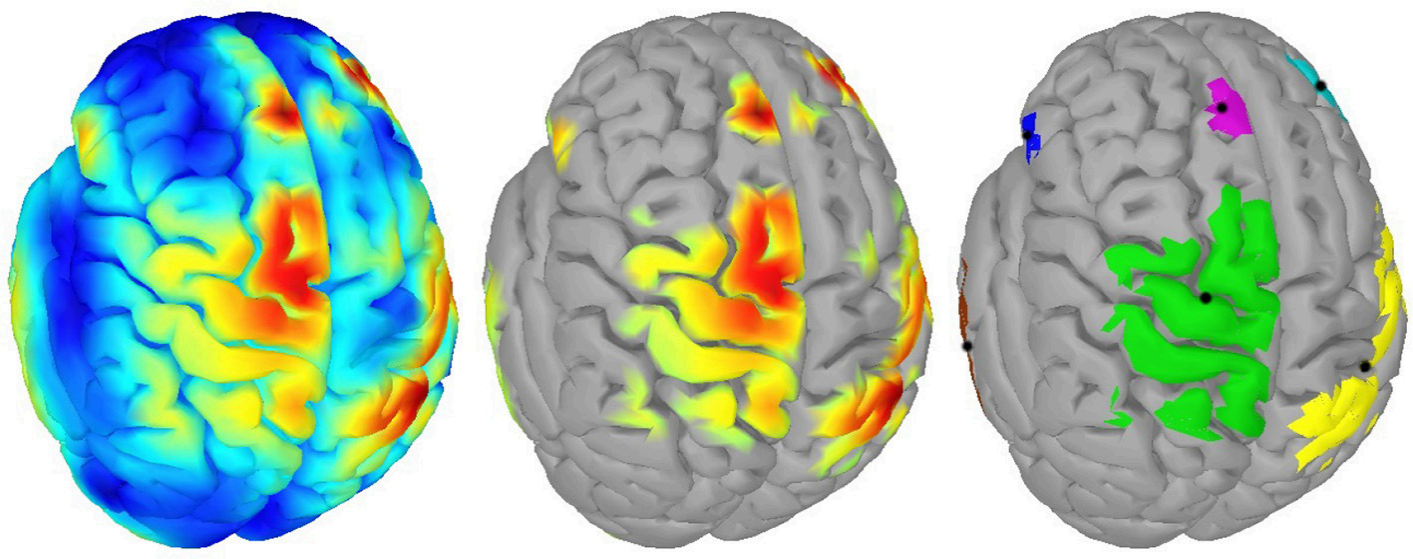
Example of clustering: **(Left)** Source activity distribution of one subject; **(Middle)** The top 5% of active sources (i.e., 750 colored vertices); **(Right)** Six computed clusters using DBSCAN (Ester et al., 1996) after hemispheres were separated. The black dots indicate the centers of mass.

For clustering 5% of the vertices in the source space were considered. These vertices, whose corresponding sources showed highest activations by taking the norm of the three directional components, were clustered using the DBSCAN algorithm (Ester et al., 1996) implemented in the Scikit-learn package (Pedregosa et al., 2011). DBSCAN is an efficient density-based clustering method that does not need the number of clusters a priori and can handle noise in the data. The algorithm has two relevant parameters: the *maximum distance* between two points for them to be considered as in the same neighborhood and the number of points in a neighborhood for a point to be considered as a core point (*minimum number of points*). For parameter setting we followed the idea that adjacent vertices should belong to the same cluster. In this way, the 95%-quantile of the distance of adjacent vertices as well as the 95%-quantile of the number of adjacent vertices were determined from the complete source space and were assigned to the maximum distance and minimum number of points, respectively. DBSCAN returns the number of clusters and their members. If points do not belong to any cluster they are collected in a specific noise cluster. No cluster centers are returned by DBSCAN. Hence, the center of mass (COM) of each cluster was computed utilizing the sources' activations as weights. An example result of DBSCAN is depicted in Figure 1. Since each hemisphere was considered separately, six distinct clusters were found.

#### 3.2.3. Normalization of the distance measure

**Figure 2:**
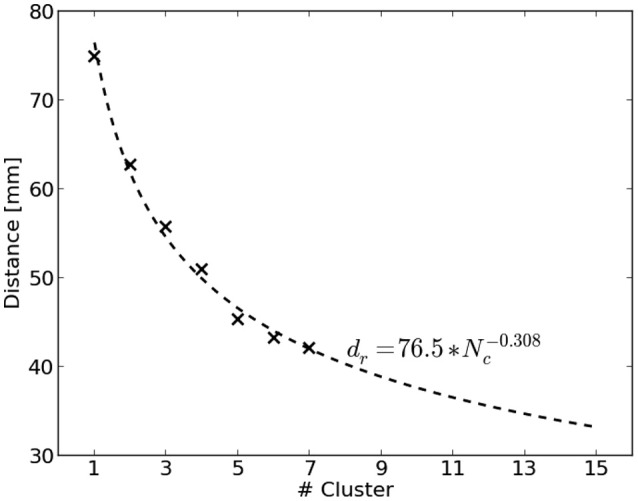
Average distance *d*_*r*_ to the reference region of randomly sampled source distributions shown dependent on the number of clusters *N*_*c*_. Two million source distributions were sampled and results are depicted as crosses. The dashed line visualizes the fitted power function $d_{r}=76.5mm*N_{c}^{-0.308}$ to extrapolate to a larger number of clusters.

The distance of the nearest COM to the reference region is biased by the total number of computed clusters for a source distribution. Figure 2 visualizes this by showing the average distance of nearest COMs obtained from two million randomly sampled source distributions in dependence of the number of clusters. It can be seen that the average distance decreases with an increasing number of clusters. To account for this bias, we used the average distance of randomly chosen source distributions as normalization factor. A power function was fit to be able to extrapolate to a larger number of clusters. Finally, the normalized distance *d*_*n*_ for a clustering *c* with *N*_*c*_ clusters was obtained by

(10) $$\begin{array}{l} d_{n}\left(c,N_{c}\right)=\frac{d\left(c\right)}{d_{r}\left(N_{c}\right)} \end{array}$$

where *d*(*c*) refers to the smallest distance of all COMs of *c* to the reference region and *d*_*r*_(*N*_*c*_) represents the average smallest distance of randomly chosen source distributions with *N*_*c*_ clusters.

Obviously it holds that the smaller *d*_*n*_ the better. Moreover, *d*_*n*_ = 1 corresponds to the same distance, a random source distribution would achieve on average.

### 3.3. Generalizations

Our approach was presented along a specific application, i.e., movement prediction of right elbow rotations, and results for this application are shown in section 4. Nevertheless, the approach can be extended and/or varied to fulfill the requirements of other applications.

For example, more than one reference region would be required to evaluate classification tasks like *left* vs. *right* or *hand* vs. *arm* movements. In these cases several reference coordinates have to be extracted from the literature. Alternatively, individual fMRI or PET recordings of the same subjects that perform the EEG-task can lead to more precise reference coordinates, but are costly. For validating the approach in this work such recordings were not available. Thus, a task with only one movement type was chosen and the analysis was restricted to a reference *region* in the primary motor cortex (section 3.2.1). However, especially in cases where the movement types of interest are somatotopically close to each other a more precise individual localization of the reference coordinates is desirable.

In addition, there are applications where in *one* condition several sources are relevant. For movement planning, this could be inside, e.g., the premotor cortex or supplementary motor area. If reliable reference regions for all sources could be obtained, an integration in the metric calculation process can be performed by summing up all nearest distances. Note that to fully assess resolution of different SLMs in case of several nearby sources, additional metrics like the spatial extent (Molins et al., 2008; Hauk et al., 2011) have to be measured. However, to obtain a reliable ground truth for the extent is even more challenging than it is for localization. Assuming that a large spatial extent would result in overlapping source activations, also the classification performance can be affected. Then, a drop in performance may correlate with a larger spatial extent of the method. This has to be investigated in the future.

### 3.4. Evaluation settings

The three SLMs were evaluated in terms of the classification performance and nearest distance to the reference region. Determining the former required a splitting into training and testing data, which was accomplished by a 3-fold cross validation. Following this setting, the distance was computed on the training data of the corresponding cross validation iteration. In this way, the suitability of the inverse operator, on which the subsequent trainable components in the processing chain will rely on, is measured. Further, the distance is evaluated on the averaged ERP-data as well as on the single-trial level.

For each metric and scale, the Wilcoxon-Signed-Rank test was performed in order to report significant differences between the SLMs. For multiple comparisons, the Bonferroni-Holm correction was applied.

## 4. Results

### 4.1. Visual comparison

**Figure 3:**
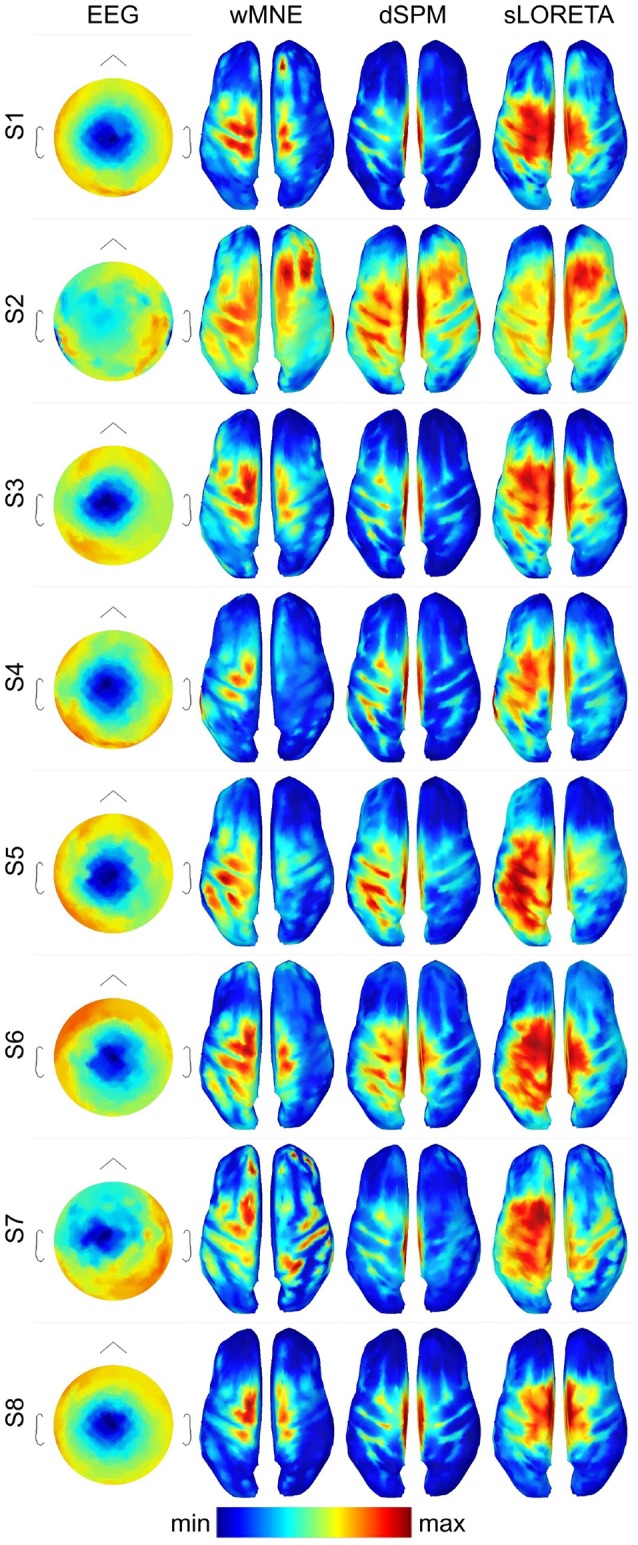
Averaged ERPs and averaged source distributions across the three training data sets for each subject. For visualization a common average reference was applied to the preprocessed EEG data, and the data is scaled to the local maxima. Source activations are depicted as the norm of the three directional components and mapped to the inflated cortex of the ICBM152 template. All plots visualize time point −0.05 s with respect to the movement onset.

For getting a visual impression of the difference between the methods refer to Figure 3. In the first column, it shows the averaged EEG data across the trials in the three training sets (3 × 80 = 240 trials) 0.05 s before movement onset for each single subject. The expected negative potential over central electrodes, i.e., the MRCP, could be observed for all subjects, although for subject S2 it was less prominent. The other three columns visualize the reconstructed source activities of the SLMs. All subjects showed at least medium activity in the left motor area. However, distribution of the highest activity values differed considerably for the three SLMs. In contrast to wMNE, dSPM often reconstructed highest activity on the centro-medial surface of the cortex. Further, in comparison to wMNE and dSPM, sLORETA revealed larger coherent areas of high activity.

### 4.2. Classification performance

In Table 3, a comparison of the three SLMs is given after integration into a movement prediction system and calculation of its performance. Here, the inverse operator was computed on the training set data and then it was applied to all single-trials of the testing data. Results show similar performance values for the majority of the subjects. But for S2, S3, and S7 differences between the SLMs could be observed. However, on average all methods obtained a similar balanced accuracy of around 88 %. Accordingly, no significant differences were found in the statistical analysis (wMNE vs. sLORETA: *p* = *n*.*s*, wMNE vs. dSPM: *p* = *n*.*s*., sLORETA vs. dSPM: *p* = *n*.*s*).

**Table 3:** Classification performance (*movement preparation* vs. *resting*) in terms of balanced accuracy.

	**wMNE**	**dSPM**	**sLORETA**
S1	**0.95 ± 0.01**	**0.95 ± 0.02**	**0.95 ± 0.01**
S2	**0.84 ± 0.01**	0.82 ± 0.02	0.76 ± 0.08
S3	**0.89 ± 0.01**	0.88 ± 0.02	0.86 ± 0.02
S4	0.90 ± 0.03	**0.91 ± 0.02**	**0.91 ± 0.02**
S5	**0.92 ± 0.01**	**0.92 ± 0.01**	0.91 ± 0.02
S6	**0.91 ± 0.02**	**0.91 ± 0.02**	**0.91 ± 0.03**
S7	0.70 ± 0.05	**0.81 ± 0.01**	0.77 ± 0.01
S8	**0.97 ± 0.01**	0.96 ± 0.02	**0.97 ± 0.01**
∅	0.88 ± 0.02	**0.89 ± 0.01**	0.88 ± 0.02

*Given are averages over the three test sets together with the standard error of the mean (SEM). The row ∅ contains mean and SEM across all individual values of the subjects. Bold values highlight best performance across SLMs*.

In comparison to our previous processing chain (e.g., Seeland et al., 2013), that did not utilize SL but the spatial filter xDAWN (Rivet et al., 2009) with 4 retained channels, performance increased on average about 2 % (mean performance and SEM without SL: 0.86 ± 0.01 BA).

### 4.3. Distance to reference region

**Figure 4:**
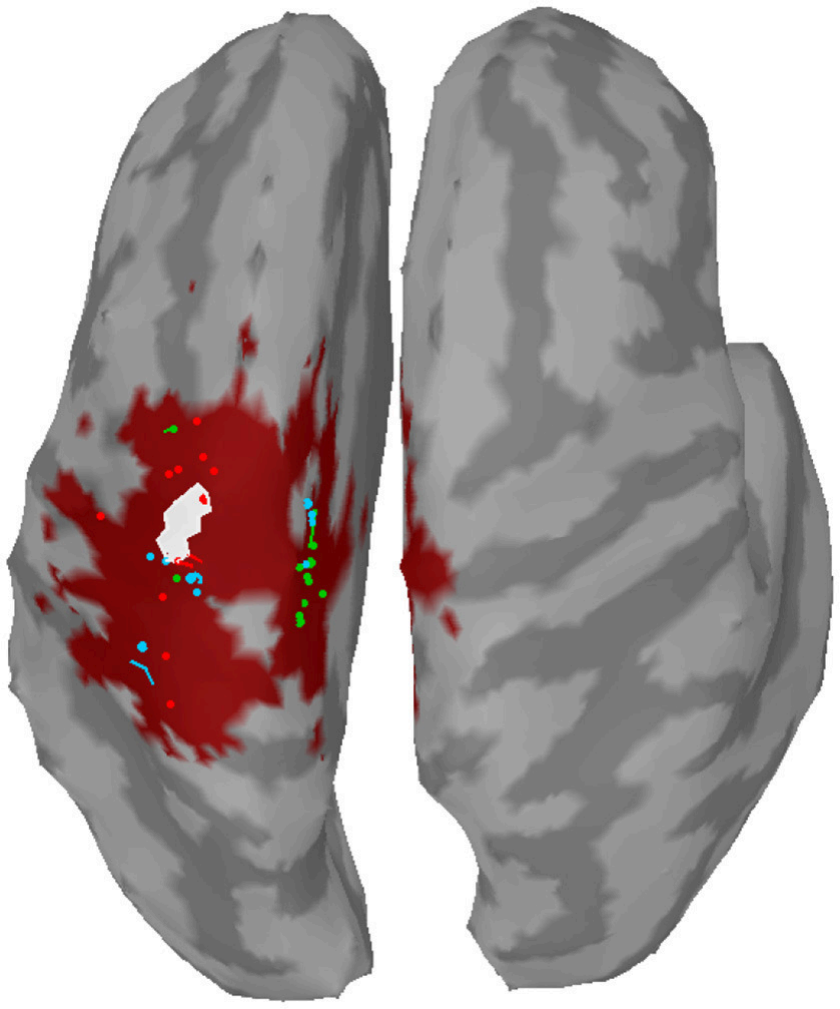
Centers of mass (points) of the nearest clusters to the reference region (white) for all subjects and training sets. Red: wMNE; Green: dSPM; Blue: sLORETA. The dark red shaded area depicts the region of highest classification performance (BA) when ~25 adjoint vertices were selected for feature extraction.

The distance to the reference region was measured based on the COM of the nearest cluster obtained from the top 5% of active sources. Figure 4 depicts all COMs together with the reference region colored in white. The reference region was hit in total seven times, once by sLORETA and six times by wMNE. Although the distribution of the COMs was rather large and suggested differces between the methods in terms of distance, the classification performance was hardly affected by the different COM positions. The dark red shaded area in Figure 4 marks a region where the classification performance is quite similar (within 2% BA). It was computed for sets of ~25 adjoint vertices that were selected for feature extraction. Table 4 lists the actual distances together with the number of found clusters averaged across the three training sets for each subject. For six of the eight subjects wMNE showed the smallest distance compared to dSPM and sLORETA. Using wMNE, on average the nearest COM was 11.05 mm away from the reference region. Approximately twice the distance, i.e., 22.30 mm, could be achieved on average with dSPM, and results using sLORETA lay in between (14.56 mm). Further, reconstructions by wMNE consisted on average in more clusters than reconstructions by the other two methods.

**Table 4:** Minimal distance to the reference region of the nearest cluster given in millimeters together with the number of computed clusters in brackets.

	**wMNE**	**dSPM**	**sLORETA**
S1	5.04 (2.7)	17.98 (3.0)	12.74 (2.0)
S2	2.20 (3.0)	17.46 (3.3)	21.26 (3.0)
S3	9.30 (3.3)	25.12 (2.0)	13.05 (2.0)
S4	37.27 (2.3)	21.97 (3.3)	11.74 (2.7)
S5	13.49 (1.7)	25.51 (2.0)	17.03 (1.7)
S6	14.91 (2.7)	21.95 (2.0)	13.41 (2.0)
S7	6.19 (5.3)	25.41 (2.3)	10.31 (2.3)
S8	0.00 (3.3)	22.98 (2.0)	16.97 (2.0)
Avg	11.05 (3.0)	22.30 (2.5)	14.56 (2.2)
SD	11.02 (1.0)	2.97 (0.6)	3.35 (0.4)

*Values are averages over the three training sets. Avg, average; SD, standard deviation*.

The influence of the number of clusters on the distance was considered by normalizing the distance with the average distance of randomly sampled source distributions with the same number of clusters. Table 5 shows the normalized minimal distances to the reference region for each subject. Results corresponding to Table 4 can be found in the upper half of Table 5. Although the number of clusters was higher for wMNE compared to the other two methods, qualitatively, results were persistent. This means, the smallest normalized distance was obtained using wMNE, followed by sLORETA and dSPM. The statistical analysis confirmed significant differences between the methods (wMNE < sLORETA: *p* < 0.0397, wMNE < dSPM: *p* < 0.0025, sLORETA < dSPM: *p* < 0.0008). Although the bias in reconstructing a source cluster in the primary motor cortex was smallest for wMNE, it should be noted that the variance was largest compared with the other two methods. The worse result of subject S4 is the reason.

In the single-trial case, i.e., when the SLM is applied to each single-trial separately, the reconstruction performed worse (lower half of Table 5). Still, wMNE was the method with least normalized distance to the reference region, but this distance almost tripled in comparison to the result on averaged data. Statistically the differences between wMNE and the other two methods were significant (wMNE < sLORETA: *p* < 3.6·10^−7^, wMNE < dSPM: *p* < 3.6·10^−11^, sLORETA vs. dSPM: *p* = *n*.*s*).

**Table 5:** Normalized minimal distance *d*_*n*_ to the reference region of the nearest cluster.

		**wMNE**	**dSPM**	**sLORETA**
Avg	S1	**0.09 ± 0.04**	0.32 ± 0.01	0.20 ± 0.00
	S2	**0.04 ± 0.02**	0.32 ± 0.05	0.38 ± 0.03
	S3	**0.17 ± 0.02**	0.40 ± 0.00	0.21 ± 0.04
	S4	0.55 ± 0.22	0.40 ± 0.03	**0.21 ± 0.11**
	S5	**0.20 ± 0.01**	0.41 ± 0.00	0.26 ± 0.01
	S6	0.26 ± 0.01	0.35 ± 0.03	**0.21 ± 0.01**
	S7	**0.14 ± 0.09**	0.42 ± 0.02	0.17 ± 0.03
	S8	**0.00 ± 0.00**	0.37 ± 0.01	0.27 ± 0.00
	∅	**0.18 ± 0.04**	0.37 ± 0.01	0.24 ± 0.02
SiT	S1	**0.35 ± 0.03**	0.58 ± 0.02	0.50 ± 0.03
	S2	**0.79 ± 0.03**	0.82 ± 0.03	0.82 ± 0.03
	S3	0.93 ± 0.04	**0.75 ± 0.02**	1.06 ± 0.03
	S4	**0.64 ± 0.03**	0.67 ± 0.02	0.79 ± 0.03
	S5	0.49 ± 0.02	0.54 ± 0.02	**0.46 ± 0.02**
	S6	**0.52 ± 0.03**	0.64 ± 0.03	0.63 ± 0.03
	S7	0.76 ± 0.02	**0.73 ± 0.02**	**0.73 ± 0.02**
	S8	**0.23 ± 0.02**	0.47 ± 0.02	0.42 ± 0.03
	∅	**0.59 ± 0.01**	0.65 ± 0.01	0.68 ± 0.01

*Given are averages over the three training sets together with the standard error of the mean (SEM). Source localization was performed on the averaged potential (Avg) as well as on the single-trials (SiT). The row ∅ contains mean and SEM across all individual values of the subjects. Bold values highlight smallest distance across SLMs*.

## 5. Discussion

In the presented work we *empirically* compared three different SLMs. Starting with a visual comparison, results showed that there are remarkable differences between the analyzed SLMs. We suggested to consider not only the classification performance, but also a distance metric to compare SLMs, knowing well that both may lead to different results. Indeed, the outstanding advantage of SLMs, that is to convert the electrode data to the brain space enabling a common frame with neuroscience research, in fact demands also an assessment of the physiological plausibility of the methods. Further, even when the classification performance is of great importance for most BCI applications, there are cases where additional criteria can be relevant. Especially in the context of robotic rehabilitation, not only the movement prediction performance might be important to trigger or control the robotic device, but also the activated brain regions during movement preparation might be used for neurofeedback or as a measure of increased cortical plasticity. In addition, in case one wants to analyze and interpret what the classifier relies on (e.g., via backtransformation Krell and Straube, 2017), it is beneficial to be able to analyze results in the brain space, i.e., to have physiologically meaningful sources of features.

In what follows we discuss our two main results, the equivalence of wMNE, sLORETA and dSPM in terms of classification performance and the significant differences in cluster distance between the three methods.

### 5.1. Comparable classification performance

As our results show, no significant differences in balanced accuracy were found between wMNE, sLORETA and dSPM for movement prediction. On the one hand several authors explained this by the similarity of the algorithms. On the other hand, we believe that the interaction with other trainable components has a substantial impact.

Hauk et al. (2011) compared MNE, dSPM and sLORETA, whereby the latter two were based on MNE and not wMNE (see also section 2.3). They showed in theory and in simulation that noise normalization has no effect on the shape of the rows of the resolution matrix *A*, if the normalization is achieved by multiplication of a diagonal matrix (like *S*_dspm_, see section 2.3.2). They further concluded that spatial filters, i.e., the rows of *G*, also only differ between those SLMs by a scalar factor. For the application in single-trial detection this means, when the same spatial filters were selected for classification, no significant differences between those SLMs can be expected. However, for practical reasons, the number of spatial filters that are passed to the feature extraction and classification has to be reduced. If the reduction process chooses different spatial filters for the different SLMs, like it was the case in our comparison, differences in performance can occur (see individual performances in Table 3). In addition, performance differences probably increase when the number of spatial filters is further reduced. In the extreme case, i.e., when only one spatial filter is selected based on the maximum source activation on the training data, we observed 0.75 (±0.03), 0.80 (±0.03) and 0.85 (±0.02) BA (± SEM) for wMNE, sLORETA, and dSPM respectively. Here, dSPM outperforms the other two methods indicating that the activation at the source with maximum activity on the training data is a better feature for classification in case dSPM is applied compared to the other two methods. This confirms that a theoretical comparison of the approaches alone might not be sufficient when it comes to application.

Although Wronkiewicz et al. (2016) asked for quantitative comparisons of distributed SLMs, they did not expect a significant influence of the specific method on the classification performance. They argue that Mosher et al. (2003) showed in a theoretical work the equivalence in the regularization strategy of several traditional SLMs, including (w)MNE and dSPM, and that the difference between the SLMs is caused by differences in the data covariance matrix. For all three methods that we compared, the noise covariance matrix was estimated in the same way (see Table 1). Given the relationship of (*W*^T^*W*)^−1^ being equal to the source covariance matrix *C*_*s*_ and the assumption that the data covariance matrix $C_{d}=LC_{s}L^{T}+C_{n}$, a difference in the analyzed methods exists between sLORETA (*C*_*s*_ = *I*) and wMNE/dSPM ($C_{d}=LC_{s}L^{T}+C_{n}$). Very recently Goel et al. (2017) also compared wMNE and sLORETA, among others, and found a significant performance decrease when normalization was applied on the inverse operator (i.e., sLORETA < wMNE). They tried to explain their results by the increased variance in the features due to normalization. Besides the fact, that they investigated different experimental paradigms (P300, ErrP & RSVP) they used a rather simple classifier of combining class conditional Gaussian probabilities across features and time. This naive Bayes approach ignores dependencies between features. On the contrary, we used a powerful SVM for classification. This algorithm might compensate for the differences between the SLMs, at least if a reasonable amount of features is used.

To summarize, all three SLM under investigation are equally suited for movement prediction. However the dependence of the results on the concrete data processing approach, including the number of features and the classifier can not be neglected. Especially in the extreme cases (i.e., low number of features or simple classifier), which are not the cases with optimal performance, differences in the SLMs that exist (e.g., *C*_*s*_, feature selection) can come to light.

### 5.2. Differences between SLMs in the distance measure

**Figure 5:**
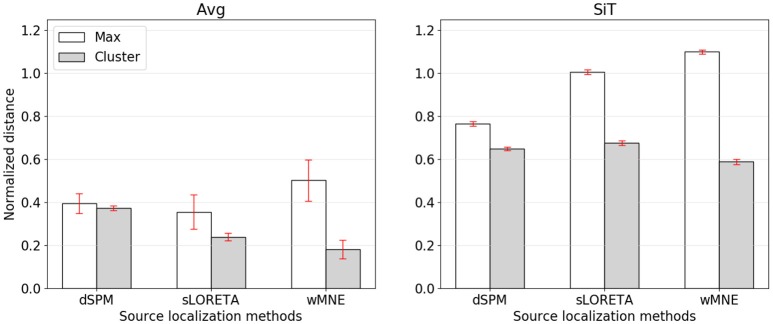
Normalized distance from the reference region to the position of maximal activity (white bars) or to the COM of the nearest cluster (gray bars). The left graph corresponds to source reconstruction on averaged data (Avg), the right to source reconstruction on single-trials (SiT). To normalize the distance to the maximum it was divided by 74.9 mm which corresponded to the average distance of randomly sampled source distributions with *one* cluster.

While the compared methods showed similar classification performances, the visual comparison as well as the cluster distance results revealed considerable differences between wMNE, sLORETA, and dSPM. For understanding the preference of wMNE, we have to look at the distance measure calculation again. In literature, most of the time distances were measured based on the source position with maximum activation (e.g., Bai et al., 2007; Molins et al., 2008; Hauk et al., 2011). In this way, the accurate reconstruction of the amplitudes is crucial. However, due to unavoidable approximations in the calculation of the inverse operator, which involves an inversion of a non-squared matrix, distributed SLMs can fail to accurately estimate the strengths of *multiple active* sources. In other words, there will always be discrepancies of the resolution matrix *A* from the ideal identity matrix that lead to amplitude estimation errors at the sources. The clustering approach introduced in this work refrained from calculating the maximum. Instead, a *set* of source positions with high activation values is considered. Yet, to see the impact of this decision, we also calculated the normalized distance to the source position with maximum activity (Figure 5). Results changed qualitatively when the distance is measured to the maximum, i.e., wMNE performed worse in comparison to dSPM and sLORETA. It can also be seen that in general the distances to the maximum were larger compared to the nearest cluster. This illustrates that on average the maximum was not part of the nearest cluster to the reference region. Instead, it was observed further away. The difference between the two distances (distance to cluster and distance to maximum) was highest for wMNE. Assuming the true maximum is located in the motor cortex (reference region), this indicates that wMNE had the greatest problems in accurately reconstructing the maximum. Further, it must be stated that we analyzed data where no artifact correction or rejection was performed. Therefore, it might happened that sources representing artifacts had higher amplitudes than sources representing the signal of interest. But a *true* maximum amplitude at a different location than the motor cortex might also be possible without artifacts, just because of the single-trial application case. When comparing average and single-trial scale in Figure 5, it is worth to mention that the normalized distance increased less for dSPM. The same effect can be observed in Table 4. Thus, dSPM appeared to be more stable than the other two SLMs under noisy conditions (single-trial, application case).

For measuring the distance in this paper, reference coordinates were extracted from the literature since individual fMRI/PET recordings were not available. To give an impression how valid our reference region is, we analyzed whether a small change in the reference region within the primary motor cortex would lead to different qualitative distance results. We obtained for the averaged as well as the single-trial case that it is possible to shift the right border of the reference region about 7.57 mm and the left border of the reference region about 28.13 mm without a different qualitative result. This supports the robustness of the obtained distance results.

If wMNE is chosen for reconstructing the sources due to our distance metric results, one has to be aware that the maximum activation is not reliable whenever interpreting amplitudes or visualizations. On average more clusters can be expected with wMNE, which may also mean more ghost sources. In addition the number of outliers and thus the variance in distance can be higher for wMNE as our results for average potentials indicate (Tables 4, 5). Therefore we suggest to use wMNE in hypothesis-driven data analyses, like for example in sensorimotor rehabilitation, where prior to analysis target regions of interest could be defined. Then the low bias in activity reconstruction in this region (e.g., a reference region in the primary motor cortex) is the advantage of wMNE.

The clustering approach required to choose additional parameters, e.g., the number of vertices to consider. This number might be motivated by an assumption about the total area that is activated during the experimental conditions. The selected 750 vertices here correspond in total to approximately 126 cm^2^ cortical surface and can cover a broad range of central and precentral areas (see also Figure 1 center for an example). If the number of vertices considered for clustering is decreased drastically the situation converges to the case where only the source position with maximum activity is considered. Conversely, if the number is drastically increased, it is more likely that all vertices will be assigned to the same cluster. The number of clusters can be additionally controlled by the *maximum distance* parameter of DBSCAN. For example, it can be seen that in several reconstructions high activity was observed not only in the motor cortex but also in the somatosensory cortex (see Figure 3). Since vertices of these two neighboring areas were assigned to the same cluster, its COM might be placed in the central sulcus. Thus, COMs in sulci (see Figure 4) were mainly not a result of high activity in these deeper brain areas, but more a result of broad high activity in neighboring areas of the sulci. In particular when comparing *distributed* SLM, these broad activations covering neighboring areas can be expected. Therefore, when areas should not belong to the same cluster, an alternative approach would be to artificially separate these regions.

In summary, we proposed a different distance calculation metric according to which wMNE outperformed sLORETA and dSPM. Due to several aforementioned reasons we believe that the cluster distance is the more appropriate distance measure for our evaluation since it is more robust against artifacts and amplitude misestimation.

## 6. Conclusion and future work

Since the primary motor cortex provides spatially relevant information, e.g., about the moving body part, SLMs are predestined for the analysis of brain activity correlated with motor functions, and for the use in movement-based BCIs. For example, in the context of stroke rehabilitation single-trial monitoring of active brain regions during therapy might increase our understanding of the recovering process. However, the explorative nature of SLM development increases, among others, the need for comparative analyses. Especially for the ease of use of those methods, application-oriented comparisons can provide useful guidance. In this paper, we suggested two criteria for comparing SLMs empirically with the aim of applying SLMs in single-trial classification. Thereby the plausibility of the reconstructed sources, and the detection performance were measured. Both metrics should be considered, e.g., when human machine interaction has to be supported in a rehabilitation scenario. The presented framework accounts for multiple active sources as well as inaccuracies in the estimation of the location of the maximum. Thus, it is specifically designed to compare distributed SLMs, but is not necessarily limited to them.

We applied our concept to the task of single-trial movement prediction. It could be shown that wMNE is superior to dSPM and sLORETA in terms of the distance between a reference region in the primary motor cortex and the next activation cluster (after a normalization procedure). Hence, if physiological plausibility is of importance wMNE should be preferred in comparison to dSPM and sLORETA. This observation only holds when working with activation clusters and not relying on single maximum activations which on average resulted in larger distances. The obtained findings could be verified by visualizations of the SLMs. This supports the feasibility of our metrics. Looking exclusively at the classification performance, all analyzed SLMs showed similar performance. Due to our choice of the processing chain this cannot be explained by the already known equality of underlying spatial filters. Instead it highlights the importance that SLMs should not only be assessed separately but also embedded in the concrete application at hand.

In the future, the interaction between SLMs and other trainable components in the processing chain will be further analyzed. For example, the generation of optimal features based on SLMs that are insensitive to inaccuracies in amplitude estimation might be promising. Further, other SLMs can be evaluated. Here, other open source software packages permitting fast usage may be most interesting for users, like FieldTrip (Oostenveld et al., 2011) or Nutmeg (Dalal et al., 2011). Last but not least, it remains to be seen how SLMs perform in other applications and classification tasks, like the differentiation between right and left movements or between different body parts.

## Author contributions

AS had the idea of the comparison, contributed to the concept, performed all analyses and wrote most of the manuscript. MK critically discussed the methodology, wrote parts of the text and revised the manuscript. SS contributed to the concept and gave valuable comments to improve the paper. EK contributed another view to the research goals, wrote part of the text and revised the manuscript. All authors gave their final approval of the version to be published and agreed to be accountable for all aspects of the work in ensuring that questions related to the accuracy or integrity of any part of the work are appropriately investigated and resolved.

### Conflict of interest statement

The authors declare that the research was conducted in the absence of any commercial or financial relationships that could be construed as a potential conflict of interest.
